# Ileocecal stent placement for malignant obstruction using a side-viewing endoscope and a balloon-equipped overtube

**DOI:** 10.1055/a-2505-9437

**Published:** 2025-01-16

**Authors:** Akihiro Maruyama, Makoto Kobayashi, Hirotaka Takeshima, Hiroshi Nakayabu, Hiroki Kato, Shintaro Tominaga, Motoyoshi Yano

**Affiliations:** 1 37036 Department of Gastroenterology, Yokkaichi Municipal Hospital, Yokkaichi, Japan

Ileocecal obstruction, particularly in malignant cases, presents unique procedural challenges in achieving optimal visualization and precise stent placement owing to the complex anatomy of this region 1 2 . Traditional forward-viewing endoscopes often have limitations, and alternative endoscopic approaches yield better outcomes in complex gastrointestinal obstructions 3 . However, reports specifically addressing malignant ileocecal obstruction are limited. Herein, we describe a successful approach to managing procedural challenges in ileocecal obstruction.

A 65-year-old man was diagnosed with cecal cancer with peritoneal dissemination that led to malignant ileocecal obstruction ( Fig. 1 ). We decided to provide palliation to improve the patient’s quality of life by relieving obstructive symptoms. Before the stenting procedure, a Gastrografin enema (Bracco, Milan, Italy) was performed ( Fig. 2 ). Considering the challenges associated with accessing the ileocecal region, we positioned an ST-CB1 overtube (Olympus, Tokyo, Japan) within the ascending colon using a forward-viewing endoscope (pCF-H290TI; Olympus), which created a stable working channel through which a side-viewing endoscope (JF-260V; Olympus) could be advanced to the obstructed site. The ST-CB1 overtube (inner diameter, 13.8 mm) accommodated the side-viewing endoscope (outer diameter, 12.6 mm), enabling precise visualization and accurate stent placement across the stricture ( Fig. 3 , Video 1 ).

**Fig. 1:**
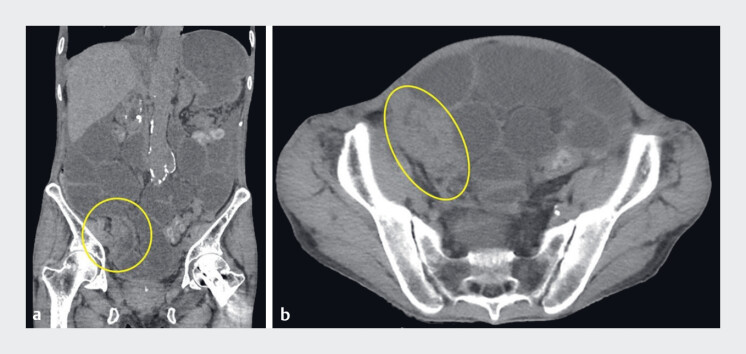
Computed tomography findings before stent placement showed thickened walls in the ileocecal region (indicated by the yellow circle), with proximal bowel dilation. **a** Coronal view. **b** Axial view.

**Fig. 2:**
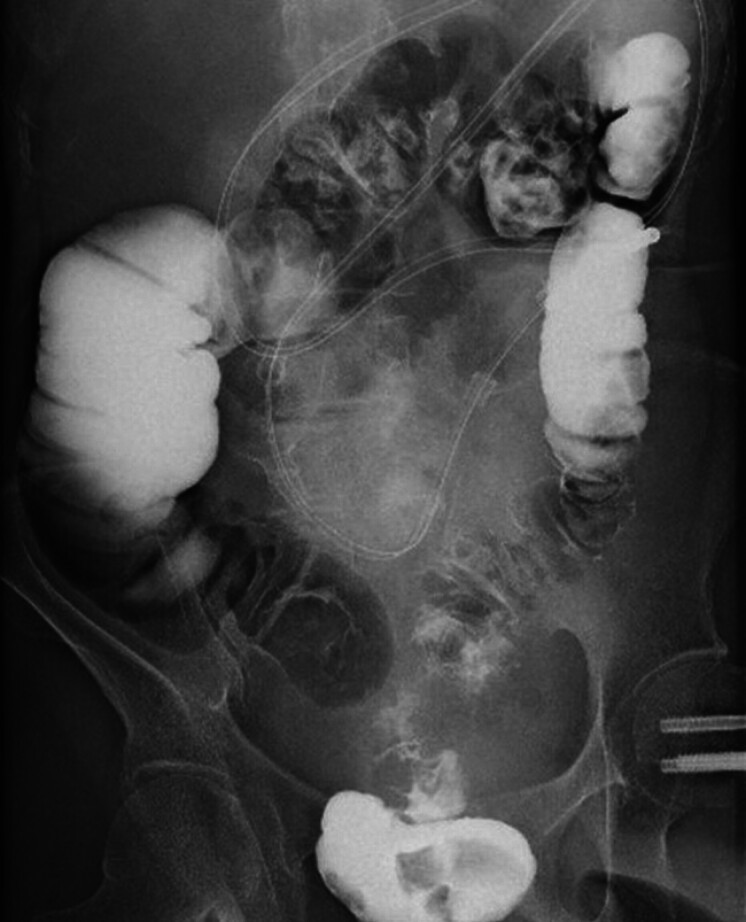
Gastrografin solution enema (Bracco, Milan, Italy), which was performed after placement of a nasoenteral ileus tube but before stent placement, showed no contrast agent flow into the ileum.

**Fig. 3:**
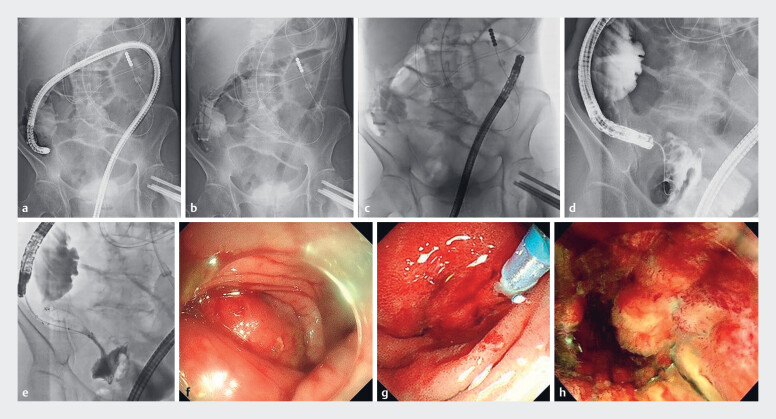
Fluoroscopic stent placement for malignant ileocecal stenosis. **a–e** Fluoroscopic images. **f–h** Endoscopic images. **a** Aided by a colonic balloon-equipped overtube (ST-CB1; Olympus, Tokyo, Japan), the pCF-H290TI scope (Olympus) was advanced to the ileocecal region. **b** The pCF-H290TI was removed, and the ST-CB1 overtube was positioned. **c** The JF-260V endoscope (Olympus) was advanced through the ST-CB1 to reach the ileocecal region. **d** A guidewire was passed through the stricture, followed by catheter advancement to allow contrast visualization on the proximal side of the stricture. **e** A colonic stent was placed (HANARO Natur Fit; Boston Scientific, Marlborough, Massachusetts, USA). **f** Visibility of the stricture was limited with the pCF-H290TI scope. **g** Stricture visibility improved using the JF-260V. **h** The HANARO Natur Fit colonic stent was placed.

Using the ST-CB1, the JF-260V endoscope was advanced into the ileocecal region. Subsequently, a 9-cm Natur Fit stent was successfully placed to address the ileocecal stenosis.Video 1

For stenting, we selected a 9-cm Natur Fit stent (Boston Scientific, Marlborough, Massachusetts, USA). Supported by a side-viewing endoscope and the ST-CB1 overtube, the stent was successfully deployed at the ileocecal obstruction site without complications ( Fig. 4 ). Post-procedure, the patient experienced significant symptomatic relief and was discharged a week later ( Fig. 5 ).

**Fig. 4:**
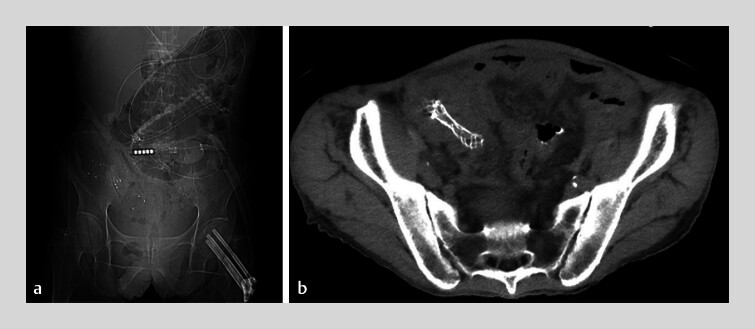
Radiographs taken 1 day post-stent placement. **a** No free air or abscess formation was observed. **b** Same-day radiograph confirmed no complications.

**Fig. 5:**
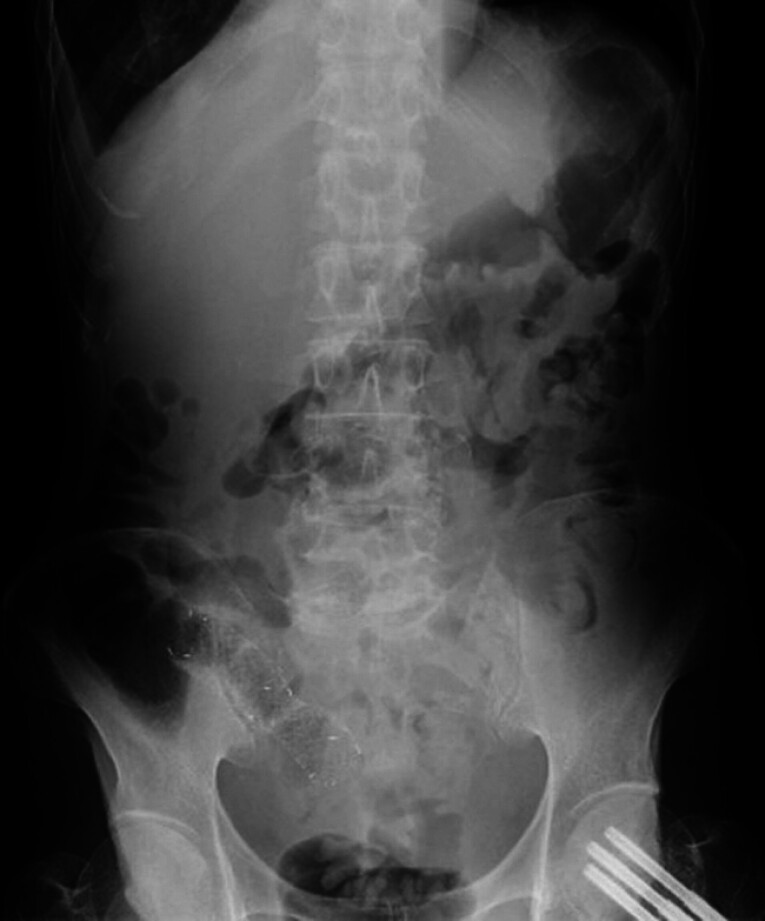
Radiographs taken 1 week post-stent placement, indicating improved bowel dilation and reduced gas accumulation.

Establishing a stable working channel with an overtube in the ascending colon facilitated visualization and control of the endoscope and optimized procedural outcomes. Additionally, the balloon component of the overtube provided the necessary anchorage, reduced scope instability, and ensured smooth maneuverability.

This approach offers a promising alternative for malignant ileocecal obstruction, particularly when conventional techniques are inadequate for visualization.

Endoscopy_UCTN_Code_TTT_1AQ_2AF

## Notes

Endoscopy E-Videos https://eref.thieme.de/e-videos
*E-Videos* is an open access online section of the journal *Endoscopy* , reporting on interesting cases and new techniques in gastroenterological endoscopy. All papers include a high-quality video and are published with a Creative Commons CC-BY license. Endoscopy E-Videos qualify for HINARI discounts and waivers and eligibility is automatically checked during the submission process. We grant 100% waivers to articles whose corresponding authors are based in Group A countries and 50% waivers to those who are based in Group B countries as classified by Research4Life (see: https://www.research4life.org/access/eligibility/ ). This section has its own submission website at https://mc.manuscriptcentral.com/e-videos .
